# Clinical results of fibroblast activation protein (FAP) specific PET for non-malignant indications: systematic review

**DOI:** 10.1186/s13550-021-00761-2

**Published:** 2021-02-19

**Authors:** Paul Windisch, Daniel R. Zwahlen, Frederik L. Giesel, Eberhard Scholz, Patrick Lugenbiel, Jürgen Debus, Uwe Haberkorn, Sebastian Adeberg

**Affiliations:** 1 Department of Radiation Oncology, Kantonsspital Winterthur, Brauerstrasse 15, 8400 Winterthur, Switzerland; 2 Department of Nuclear Medicine, Heidelberg University Hospital, Heidelberg, Germany; 3 Department of Cardiology, Heidelberg University Hospital, Heidelberg, Germany; 4 Heidelberg Center for Heart Rhythm Disorders (HCR), Heidelberg University Hospital, Heidelberg, Germany; 5 DZHK (German Centre for Cardiovascular Research), Partner Site Heidelberg/Mannheim, University of Heidelberg, Heidelberg, Germany; 6 Department of Radiation Oncology, Heidelberg University Hospital, Heidelberg, Germany; 7 Heidelberg Institute of Radiation Oncology (HIRO), Heidelberg, Germany; 8 National Center for Tumor Diseases (NCT), Heidelberg, Germany; 9 Clinical Cooperation Unit Radiation Oncology, German Cancer Research Center (DKFZ), Heidelberg, Germany; 10 Heidelberg Ion-Beam Therapy Center (HIT), Department of Radiation Oncology, Heidelberg University Hospital, Heidelberg, Germany; 11 German Cancer Consortium (DKTK), Partner Site, Heidelberg, Germany; 12 Clinical Cooperation Unit Nuclear Medicine, German Cancer Research Center (DKFZ), Heidelberg, Germany; 13 Translational Lung Research Center Heidelberg (TLRC), German Center for Lung Research (DZL), Heidelberg, Germany

**Keywords:** Fibroblasts, Fibroblast activation protein, FAP, Positron emission tomography, PET, PET-CT

## Abstract

**Purpose:**

Small molecules targeting fibroblast activation protein (FAP) have emerged as a new group of tracers for positron emission tomography (PET) in 2018. While most of the existing literature has been focussed on the application of FAP-specific PET in various kinds of cancers, some researchers have, both intentionally or unintentionally, used FAP-specific PET in patients with non-cancerous diseases. The purpose of this systematic review is therefore to summarize the available evidence of FAP-specific PET for non-malignant indications.

**Methods:**

The MEDLINE database was searched for studies presenting the clinical use of FAP-specific PET, the records were screened according to PRISMA guidelines and articles containing patients suffering from non-malignant diseases were included.

**Results:**

Sixteen studies with 303 patients were included. FAP-specific PET has been used in cardiac imaging, IgG_4_-related disease, benign tumors as well as various kinds of inflammation. Two prospective studies on FAP-specific PET for IgG_4_-related disease show its potential to differentiate inflammatory from fibrotic lesions, which could be used to determine the management of these patients.

**Conclusion:**

While publications on FAP-specific PET for non-malignant indications are mostly limited to case reports and incidental findings, the first retrospective and prospective studies present promising results for IgG_4_-related as well as cardiovascular disease that warrant further research. Several currently recruiting trials will add to the body evidence in the next few years.

## Introduction

The development of small molecules targeting fibroblast activation protein (FAP) as tracers for positron emission tomography (PET) in 2018 has sparked considerable interest mainly in the oncological community [1, 2].

FAP has dipeptidyl peptidase as well as endopeptidase activity and its expression is limited on normal adult tissue. The expression can, however, increase significantly during tissue modelling, wound healing as well as in diseases such as arthritis, atherosclerosis and different cancers.

While most studies therefore evaluate the application of FAP-specific PET in patients with various kinds of cancers, the use of FAP-specific PET in non-malignant indications has initially been limited to occasional case reports [3].

However, as studies on FAP tracers in cancer began to grow in number and size, the amount of incidental findings of non-malignant diseases began to increase [4]. In addition, the promising results from the early case reports have caused the first dedicated studies on FAP-specific PET for non-malignant indications to be conducted which in turn have been met with more interest [5, 6].

Herein, we therefore conducted a systematic review of the use of FAP-specific PET in non-malignant indications to summarize the available evidence, to indicate areas where the available evidence is limited and to highlight interesting case reports that might inspire future research.

## Methods

The review was conducted according to the PRISMA guidelines as applicable [7]. Studies published in English not earlier than 2018 that used FAP-specific PET in humans for any kind of non-malignant indication, either intentionally or unintentionally, were included. No limits regarding size of the patient collective or length of follow-up were applied. The MEDLINE database was searched on September 16^th^ 2020 via the freely accessible PubMed interface. The query was designed to show results whose titles contained either of the words “fibroblast”, “FAP” or “FAPI” in combination with either “positron”, “PET” or “Imaging” (example syntax: “((Fibroblast[Title]) OR (FAP[Title]) OR (FAPI[Title])) AND ((PET[Title]) OR (Positron[Title]) OR (Imaging[Title]))”). After exclusion of duplicates, the titles were screened and only original research publications proceeded to full-text screening. All articles that did not contain any patients suffering from non-malignant disease or did not provide any information in addition to the presence of the disease were excluded. Risk of bias in individual studies was assessed by gathering the conflict of interest (COI) with a concrete relation to the submitted work and funding statements as reported in each publication.

## Results

**Fig. 1:**
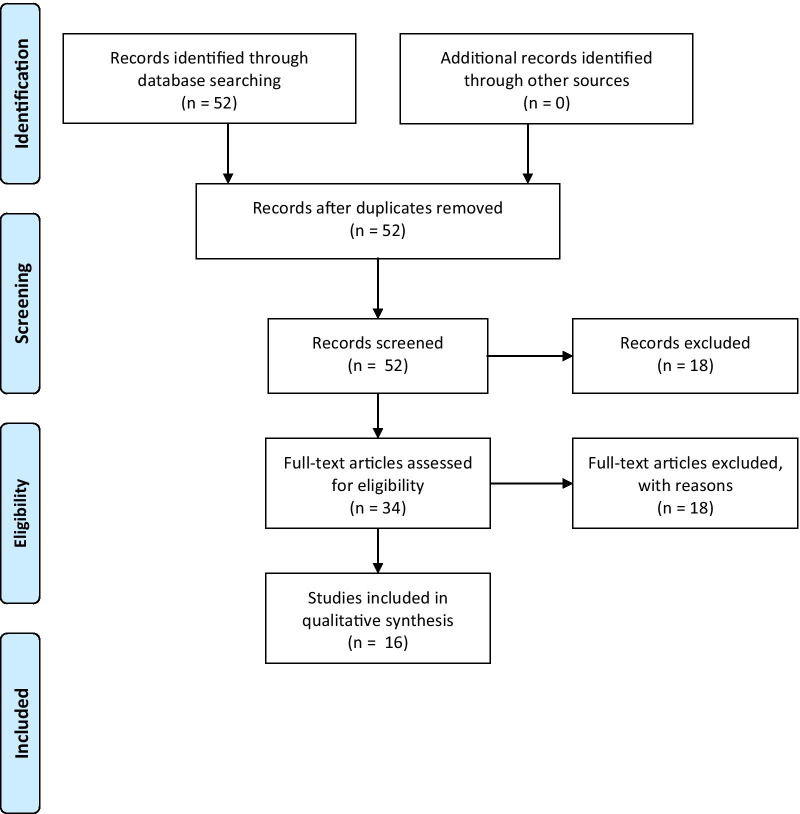
Workflow of the literature search according to PRISMA guidelines

The inclusion workflow is depicted in Fig. 1. The query returned 52 publications and no duplicates. During screening of the records, 18 results were excluded due to presenting only preclinical results [2, 8–17], being a dataset [18] or a commentary/editorial [19–24]. During screening of the full-text articles, 18 articles were excluded as they only provided information on FAP-specific PET in cancer patients [25–42]. Ultimately, 16 articles were included [4–6, 43–55] whose characteristics as well as the respective number of patients are depicted in Table 1.

**Fig. 2:**
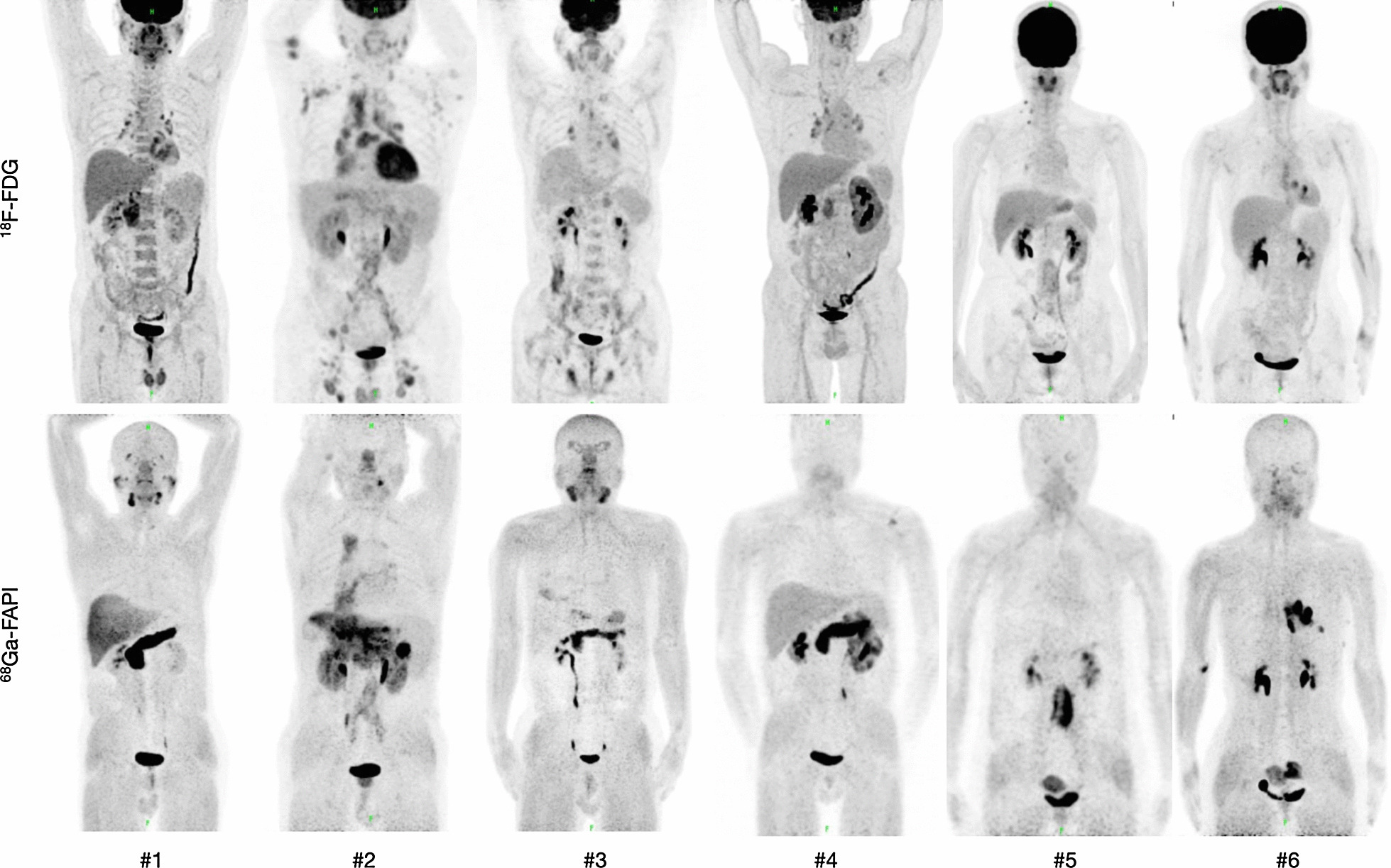
Comparison of 6 patients with IgG_4_-related disease (IgG_4_-RD) undergoing FDG and FAP-specific PET. FAP-specific PET detected IgG_4_-RD in the pancreas (patient #1, 2, 3, 4) bile duct/liver (patient #1, 2, 3), retroperitoneal fibrosis (patient #5), lung/pleura (patient #6), and salivary gland (patient #1, 3). Positive Iymph nodes on FDG PET show no enhancement on FAP-specific PET (patient #2, 3) (Fig. 2). This research was originally published in JNM. Luo et al. Fibroblast activation protein targeted PET/CT with ^68^Ga-FAPI for imaging IgG_4_-related disease: comparison to ^18^F-FDG PET/CT. J Nucl Med. 2020. ©SNMMI [47]

**Table 1:** Summary of studies on FAP-specific for non-malignant indications

Author	Year	Non-malignant Indication (patients)	Tracer (patients)	Key findings	COI	Funding
Heckmann et al. [5]	2020	Investigation of cardiovascular disease (229 patients)	Not specified (229 patients)	High signal intensities are correlated with cardiovascular and metabolic risk factors (arterial hypertension, diabetes mellitus, obesity) Focal enrichment patterns might be suggestive of underlying cardiovascular disease	Patent application	Fellowship of the German Center for Cardiovascular Research
Xu et al. [43]	2020	Shoulder arthritis (1 patient)	^68^Ga-FAPI-04 (1 patient)	FAPI-04 showed a strong uptake in the previously known arthritis	None	None
Shi et al.[44]	2020	Dilated cardiomyopathy/chronic heart failure (1 patient)	^68^Ga -FAPI-04 (1 patient)	FAPI-04 showed an increased, heterogeneous uptake pattern The strongest uptake was noted in the left ventricular inferior wall	None	None
Schmidkonz et al. [6]	2020	IgG_4_-related disease (27 patients)	^68^Ga -FAPI-04 (27 patients)	FAP-specific PET can visualize fibrosis in the absence of inflammation and therefore differentiate fibrotic from inflammatory activity	Patent application	German Research Foundation, European Research Council Starting Grant, ELAN and IZKF Fonds, Wilhelm Sander Stiftung, Federal Ministry of Education and Research
Hayrapetian et al. [45]	2020	Elastofibroma dorsi (1 patient)	^68^Ga -FAPI-46 (1 patient)	FAPI-46 showed a moderate yet more intense uptake compared to FDG in an infrascapular elastofibroma dorsi	Equity in Sofie Biosciences	Molecular Imaging Research Grant for Junior Academic Faculty, Philippe Foundation Inc, ARC Foundation
Hao et al.[46]	2020	Tuberculosis (1 patient)	^68^Ga -FAPI-04 (1 patient)	FAPI-04 showed an increased uptake in tuberculous lesions in brain, lung and spine compared to FDG	None	None
Chen et al.[4]	2020	Tuberculosis (2 patients) Pancreatitis (1 patient) Post-treatment inflammatory reactions (2 patients) Esogastritis (2 patients) Hepatic adenoma (1 patient) Pancreatic cystadenoma (1 patient)	^68^Ga -FAPI-04 (9 patients)	FAPI-04 showed an increased uptake in tuberculosis, pancreatitis and post treatment inflammatory reaction No increased uptake could be found in esogastritis, hepatic adenoma and pancreatic cystadenoma	None	National Natural Science Foundation of China, key medical health projects in Xiamen
Luo et al.[47]	2020	IgG_4_-related disease (26 patients)	^68^Ga -FAPI-04 (26 patients)	FAPI-04 showed low background in the head and neck and was therefore more able to detect IgG_4_-related disease in this region	None	CAMS Innovation Fund for Medical Sciences
Shi et al.[48]	2020	Benign hepatic nodules with granulation tissue (1 patient)	^68^Ga -FAPI-04 (1 patient)	The only benign hepatic tumor in the study showed a negligible uptake	None	National Natural Science Foundation of China, Fundamental Research Funds for the Central Universities, CAMS Innovation Fund for Medical Sciences
Pang et al.[49]	2020	Possible hormonotherapy-induced chronic inflammation of the adrenals (1 patient)	Not specified (1 patient)	FAP-specific PET showed bilateral enhancement of the adrenals in a patient on total androgen blockage	None	None
Zhao et al.[50]	2020	Hepatic adenoma (1 patient)	Not specified (1 patient)	FAP-specific PET showed low enhancement in three benign hepatic nodules in a patient with liver cirrhosis	None	None
Chen et al.[51]	2020	Benign pleural nodules (1 patient)	Not specified (1 patient)	FAP-specific PET showed low enhancement in benign pleural nodules but a focally increased uptake in a lung nodule which turned out to be a primary lung adenocarcinoma	None	None
Pan et al.[52]	2020	IgG_4_-related disease (1 patient)	Not specified (1 patient)	While FAP-specific PET showed uptake in regions that were negative on FDG-PET such as the lacrimal glands, most FDG-avid lymph nodes were negative	None	CAMS Initiative for Innovative Medicine
Luo et al.[53]	2020	Tumor-induced pancreatitis (1 patient)	Not specified (1 patient)	FAP-specific PET showed intense uptake of an enlarged pancreas with pancreatitis masking the presence of pancreatic cancer as the underlying cause	None	CAMS Initiative for Innovative Medicine
Totzeck et al.[51]	2019	Coronary artery disease/possible cardiotoxicity (1 patient)	Not specified (1 patient)	FAP-specific PET showed intense uptake in the left ventricle in a cancer patient with a history of coronary artery disease and chemotherapy but no current acute or chronic coronary syndromes	None	None
Luo et al.[55]	2019	IgG_4_-related disease (1 patient)	Not specified (1 patient)	While FAP-specific PET showed uptake in regions that were negative on FDG-PET such as the pancreas, most FDG-avid lymph nodes were negative	None	CAMS Initiative for Innovative Medicine

COI, conflict of interest

### Sources of bias

A conflict of interest (COI) related to the submitted work was present in three publications (19%). A patent application for quinoline-based FAP targeting agents for imaging and therapy in nuclear medicine was the most frequent COI and present in two publications (13%). A funding source was named in nine publications (56%). The most frequent funding source was the Chinese Academy of Medical Sciences (CAMS) Initiative for Innovative Medicine in five publications (31%).

### Cardiac imaging

Totzeck et al. published the first case report on cardiac imaging using FAP-specific PET in 2019 [54]. The patient previously diagnosed with metastasized pancreatic adenocarcinoma had a history of coronary artery disease and had received several systemic antineoplastic therapies (gemcitabine, Nab-Paclitaxel, modified FOLFIRINOX). Though he presented without signs of acute or chronic coronary syndromes, FAP-specific PET showed a strong uptake of the left ventricular myocardium which at that time had an ejection fraction of 41%. The authors hypothesize that this enhancement might be a possible sign of FAP activation due to cardiotoxic tissue damage.

Shi et al. performed FAP-specific PET on a patient with nonischemic chronic heart failure (CHF) induced by inflammation and fibrosis activation [44]. The strongest uptake was found in the left ventricular inferior wall and the left atrium (SUV_max_ = 2.60 and 2.39 respectively). The uptake in the right ventricle and atrium was slightly less (SUV_max_ = 2.10). The ejection fraction (EF) was slightly better for the right ventricle (left ventricular ejection fraction = 12.7%, right ventricular ejection fraction = 18.2%).

The first study that retrospectively analyzed the activity of FAP in the hearts of a larger collective of patients was published by Heckmann et al. and comprised a total of 229 patients with metastasized cancer in a modeling (185 patients) and a confirmatory cohort (44 patients) [5]. In addition to undergoing FAP-specific PET, patients were screened for multiple cardiovascular risk factors, cardiac medication, antineoplastic systemic therapies and prior radiotherapy to the chest. The multivariate modelling found an association between an increased uptake on FAP-specific PET and a hypothyroid metabolic state, overweight, diabetes mellitus as well as prior radiotherapy to the chest. Focal accumulation of the tracer was associated with coronary artery disease, the presence of cardiovascular risk factors and aspirin intake. While the uptake in patients with only a single cardiovascular risk factor was limited, the combination of risk factors caused a pronounced increase. In addition, a patient scanned while currently undergoing radiotherapy to the thorax showed a strong increase in myocardial tracer uptake.

### IgG_4_-related disease

Luo et al. published a case report of a patient with IgG_4_-related disease (IgG_4_-RD), a systemic inflammation associated with the infiltration of IgG_4_-secreting plasma cells in the inflamed tissue, who underwent FAP-specific PET in 2019 [55]. On FDG PET, the patient exhibited enhancement in multiple lymph nodes in the head and neck as well as the mediastinum, the right lung, the parotid and submandibular gland. While FAP-specific PET also showed enhancement in the submandibular and parotid gland as well as the pulmonary nodules, the FDG-avid lymph nodes were negative but instead there was uptake in the uncinate process of the pancreas.

A case report by Pan et al. reported similar findings regarding FAP-negative FDG-avid lymph nodes, but an enhancement in the lacrimal glands that had not been spotted on FDG PET [52].

Two studies then compared FDG to FAP-specific PET in a prospective setting:

Luo et al. recruited 26 patients with IgG_4_-RD who underwent both PET imaging with both tracers within one week [47]. The study confirmed the propensity of FAP-specific PET to show involvement in the pancreas, bile duct, liver and lacrimal glands while FDG-avid lymph nodes were mostly negative.

The most recent study by Schmidkonz et al. enrolled 27 patients with IgG_4_-RD who underwent FDG and FAP-specific PET within two days [6]. In addition, a pathologist scored the extent of inflammation and fibrosis from previous biopsies using a semi-quantitative scale. Seven patients underwent their PET/CTs within two days prior to the initiation of rituximab therapy and repeated them within two days of therapy completion roughly seven months later. While lesions whose histopathology results showed strong lymphoplasmacytic infiltration of IgG_4_-positive cells had a stronger uptake on FDG PET, lesions with lots of activated fibroblasts expressing FAP were more positive on FAP-specific PET. In addition, antiinflammatory therapy showed only a partial reduction of enhancement in fibrotic lesions.

### Tuberculosis

Hao et al. published a case report on a patient with a history of pulmonary tuberculosis and previous anti-tuberculous therapy [46]. When the patient presented with headache, lower back pain as well as limited mobility, a brain MRI revealed multiple enhancing lesions and Mycobacterium tuberculosis was found in the cerebrospinal fluid. FDG PET showed hypermetabolic lesions in the lumbar spine and hilar lymph nodes. Due to high background enhancement of the brain tissue, the lesions discovered on MRI were not visible. FAP-specific PET showed lesions in both lungs, the lower abdomen, lower spine and brain consistent with the findings from the MRI.

In a study by Chen et al., FAP-specific PET was conducted for a variety of indications with inconclusive findings on FDG PET [4]. The two patients with tuberculosis also exhibited a strong uptake in several lesions.

### Benign tumors

The description of benign tumors on FAP-specific PET has been limited to occasional cases.

Chen et al. published a case report on a patient with a history of rectal cancer who underwent FDG PET prior to surgery which showed multiple pleural nodules as well as a solitary pulmonary nodule in the left lung with low uptake [51]. FAP-specific PET showed equally low uptake in the pleural nodules but an increased uptake in the pulmonary nodule. A subpleural nodule was biopsied but diagnosed as benign while the pulmonary nodule was found to be a primary adenocarcinoma of the lung.

Zhao et al. published a case report on a patient with cirrhosis and three liver nodules that were found on contrast-enhanced MRI but not visible on FDG PET [50]. While the cirrhotic liver showed an increased background on FAP-specific PET, the nodules showed only low activity and were therefore clearly visible. One of the nodules was diagnosed via biopsy as hepatic adenoma and none of the nodules showed change on a follow-up MRI at three months.

Shi et al. conducted a study on 17 patients presenting with suspicious hepatic lesions on CT, MRI or ultrasound and ultimately underwent surgery or biopsy [48]. The only patient who was confirmed to have ‘benign nodules with granulomatous tissue’ presented with negligible uptake (SUV_max_ = 0.72). The same applies to patients with hepatic adenoma and pancreatic cystadenoma in the study by Chen et al. [4].

The only publication that showed a moderate uptake for a benign tumor is a case report by Hayrapetian et al. on an infrascapular Elastofibroma dorsi in a patient with esophageal cancer [45].

### Other inflammation

Luo et al. published a case report on a patient with pancreatic cancer and tumor-associated pancreatitis [53]. While FDG PET revealed a nodular lesion in the uncinate process, FAP-specific PET showed intense activity in the whole enlarged pancreas masking the lesion that was later confirmed as pancreatic ductal carcinoma.

Pang et al. published a case where FAP-specific PET was able to detect gastric signet cell carcinoma in a patient with a history of prostate cancer [49]. The authors note a bilateral uptake in the adrenal glands which they hypothesize to be hormonotherapy-induced chronic inflammation associated with androgen blockage.

The study by Chen et al. describes high uptake in another patient with pancreatitis and two patients with unspecified “post-treatment inflammatory reaction” but low uptake in two patients with esogastritis [4].

Xu, Zhao, Ding et al. presented a patient with prostate cancer with known arthritis of the left shoulder that also showed intense uptake on FDG as well as FAP-specific PET [43].

## Discussion

As FAP-specific PET is a fairly novel imaging modality, the number of prospective studies is still low.

The cases where FAP-specific PET has been used in patients with benign tumors hint at a low tracer uptake. However, benign tumors that contain a large amount of active fibroblasts can exhibit stronger enhancement which is illustrated by the patient with elastofibroma dorsi [45]. Larger, prospective studies are warranted to provide information regarding the accuracy of FAP-specific PET for differentiating malignant from benign and potentially fibrotic lesions in different regions of the body.

Since even the role of FDG PET in tuberculosis is still being discussed, the cases of FAP-specific PET for tuberculosis are insufficient to draw meaningful conclusions. While FAP-specific PET could have an advantage in areas such as the brain where FDG PET shows a high background enhancement, the uptake of FDG in tissue with increased metabolism could be beneficial to reveal new lesions that have not yet undergone fibrotic remodelling. A head-to-head comparison between FAP-specific PET and FDG, ideally with follow-up imaging after the application of anti-tuberculous therapy, could help to clarify the role of FAP-specific PET in this disease. The same applies to other tracers that have been tested for this indication without demonstrating clear superiority or inferiority such as ^68^Ga-citrate [56].

The fact that FAP-specific PET can show a strong uptake in inflamed tissue has been viewed as a possible obstacle for its application in oncology since determining the extent of a tumor can be difficult when the surrounding tissue is inflamed which is demonstrated by the case report of the patient with tumor-associated pancreatitis [53]. Since increased metabolism is thought to precede the recruitment of activated fibroblasts, future studies could investigate if there is a possible application for FAP-specific PET to differentiate between acute and chronic inflammation and thereby predict the response to antiinflammatory therapy.

In IgG_4_-RD, FAP-specific PET was able to identify lesions characterized by fibrosis that subsequently responded worse to antiinflammatory therapy compared to lesions that were characterized by inflammation. If adequate treatment options were available for both types of lesions, FAP-specific PET could be used in a trial setting to determine whether it is able to improve the management of these patients.

In cardiac imaging, FAP-specific PET showed an association with various risk factors, especially in combination with each other. Similar results regarding an association of FAPI uptake with coronary artery disease, age and left ventricular ejection fraction were obtained in another retrospective study by Siebermair et al. on 32 cancer patients [57]. Together with preclinical data highlighting the role of FAP in myocardial infarction and FAP-specific PET of small animals, the application of FAP-specific PET in humans might provide more insights into the role of fibroblasts in acute as well as chronic heart disease [17, 58, 59]. Another question of interest could be whether the cardiac FAP signal intensity can serve as another prognostic, maybe even predictive, biomarker in addition to the already established cardiovascular risk factors.

Establishing the role of FAP-specific PET would also benefit from comparisons to other tracers that are mostly being investigated in the role of acute myocardial infarction such as the CXCR4 ligand ^68^Ga-pentixafor [60–62].

The findings that prior radiotherapy to the chest was associated with increased uptake and that a patient currently undergoing radiotherapy to the chest showed an even stronger uptake are particularly interesting from a radiation oncology perspective to better understand and potentially manage treatment-related side effects..

While it has been known for several decades that higher doses to the heart of 30 Gy or more can cause cardiotoxicity within the first years following radiotherapy, investigating the effects of lower doses to the heart is considerably more difficult as the latency period between the application of radiotherapy and the development of heart disease is longer [63]. Radiation-related heart disease can manifest itself as pericarditis, pericardial fibrosis, myocardial fibrosis as well as coronary artery disease and several cardiac avoidance techniques such as heart blocks, prone breast boards and respiratory gating methods have been implemented to reduce its incidence. Being able to non-invasively analyze the activation of fibroblasts during or after radiotherapy could provide more insight into the pathophysiology of radiation-related heart disease and in turn enable radiation oncologists to tailor their therapy to the individual patient. Since sparing organs at risk in close proximity to the target volume requires compromise to the target coverage or the constraints of other organs at risk, one might, for example, investigate if cardiac toxicity could also be reduced by sparing parts of the heart that already show increased fibroblast activation prior to radiotherapy instead of the heart as a whole.

Considering that radiotherapy is a mainstay of treatment for breast cancer as well as in functionally inoperable patients and that many systemic therapies have cardiotoxic side effects as well, progress in the understanding of radiation-related heart disease has the potential to improve outcomes for many patients [64].

With the expanding application of stereotactic body radiation therapy (SBRT) for smaller primary tumors of the lung or in the oligometastatic setting, this potential is likely to increase [65].

In addition to the heart, FAP-specific PET could be used to image fibrotic activity in a variety of organs. A case report by Sonni et al. describes a 36-year-old patient with cervical cancer who underwent FAP-specific PET that showed “symmetric, diffuse, peripheral bilateral breast uptake” that the authors hypothesize to be caused by hormonal stimulation since the patient had recently received gonadotropin injections for oocyte retrieval [66].

The capability of FAP-specific PET to detect fibrotic remodelling non-invasively and potentially earlier than other imaging modalities could be used in a variety of different organs and diseases. Future studies could try to assess whether FAP-specific PET at baseline is better at predicting radiation pneumonitis than conventional pretreatment imaging. In addition, FAP-specific PET might be used to monitor other diseases that are primarily characterized by inflammation and/or fibrotic remodelling such as systemic sclerosis and vasculitis.

In addition to imaging and monitoring disease, molecules targeting FAP have always been of interest for delivering therapies in a targeted fashion. While this interest has historically been focussed on oncology, bringing antiinflammatory drugs to the areas that are the most affected by inflammation and subsequent fibrosis could provide additional treatment options in non-malignant diseases as well by limiting side effects associated with the systemic distribution of antiinflammatory drugs.

Possible limitations at study level include the presence of a patent and/or equity COI in several of the included publications. A possible limitation at review level is that the search was limited to the MEDLINE database which is, however, mitigated by the fact that FAP-specific tracers are currently only used by a limited number of research groups whose results are published in MEDLINE-indexed journals. Another limitation is that the total number of patients who received FAP-specific PET for a given indication cannot be determined exactly as it cannot be excluded that some publications from the same group contain at least a subset of patients that is analyzed more than once. Lastly, co-authors of this review are in part represented as co-authors on one of the included articles [5].

As of September 2020, searching clinicaltrials.gov yielded five recruiting or not yet recruiting prospective trials on FAP-specific PET for non-malignant indications (Table 2). Four studies on rheumatoid arthritis, inflammatory bowel disease, IgG_4_-RD and Crohn’s disease are estimated to be completed by October 2021, while another trial on FAP-specific PET in liver fibrosis is estimated to be completed by December 2023.

**Table 2:** Recruiting and not yet recruiting trials for FAP-specific PET for non-malignant indications on clinicaltrials.gov

Title	Estimated enrollment	Estimated Study completion Date	Indication	Tracer	Location	Key Interventions
Characterizing Rheumatoid Arthritis With ^68^Ga-FAPI PET/CT	100 patients	10/2021	Arthritis	Not specified	Peking Union Medical College Hospital	Patients who underwent FDG PET for suspected or confirmed untreated arthritis will receive and additional FAP-specific PET within two weeks
Characterizing Inflammatory Bowel Disease With ^68^Ga-FAPI PET/CT	100 patients	10/2021	Inflammatory bowel disease	Not specified	Peking Union Medical College Hospital	Patients who underwent FDG PET for suspected or confirmed untreated inflammatory bowel disease will receive and additional FAP-specific PET within two weeks
Characterizing IgG_4_-RD With ^68^Ga-FAPI PET/CT	100 patients	10/2021	IgG_4_-related disease	Not specified	Peking Union Medical College Hospital	Patients who underwent FDG PET for suspected or confirmed untreated IgG_4_-related disease will receive and additional FAP-specific PET within two weeks
^68^Ga-FAPI PET/CT in Liver Fibrosis Patients (GFAPILF)	50 patients	12/2023	Liver fibrosis	^68^Ga -FAPI-04	First Affiliated Hospital of Fujian Medical University	Patients with suspected newly diagnosed or previously treated liver fibrosis will receive FAP-specific PET, transient elastography and blood testing
^18^F-FDG and ^68^Ga -FAPI PET/CT in Crohn's Disease	30 patients	09/2021	Crohn’s disease	Not specified	Peking Union Medical College Hospital	Patients with Crohn’s disease with proofs of intestine stricture by other modalities (MRI/CT/ultrasound/endoscopy) will undergo FDG and FAP-specific PET within two days In patients with resection of the stenotic intestine, the extent of inflammation and fibrosis will be analyzed

## Conclusion

While the research on FAP-specific PET for non-malignant indications is still in the stage of generating hypotheses and not changing practice, the studies that have been published show promising results that warrant further research, especially in cardiac imaging and immunology/rheumatology. Several studies on FAP-specific PET for non-malignant indications will provide additional evidence in the next few years.

## Data Availability

All included manuscripts are provided in Table 1. The results of the PubMed query are provided as a txt-file in the supplement.
